# Microscopic and molecular aspects of skeletal muscle alterations in cerebral palsy

**DOI:** 10.1111/dmcn.70044

**Published:** 2025-11-09

**Authors:** Sebastian Edman, Oscar Horwath, Eva Pontén, Sudarshan Dayanidhi, Ferdinand von Walden

**Affiliations:** ^1^ Division of Pediatric Neurology, Department of Women's and Children's Health Karolinska Institutet Stockholm Sweden; ^2^ Molecular Muscle Physiology and Pathophysiology Group, Department of Physiology and Pharmacology Karolinska Institutet Stockholm Sweden; ^3^ Department of Physiology, Nutrition and Biomechanics The Swedish School of Sport and Health Sciences Stockholm Sweden; ^4^ Department of Pediatric Orthopaedic Surgery Karolinska University Hospital Stockholm Sweden; ^5^ Shirley Ryan AbilityLab Chicago IL USA; ^6^ Feinberg School of Medicine Northwestern University Chicago IL USA

## Abstract

Cerebral palsy (CP), the most prevalent childhood‐onset motor disability, frequently entails progressive musculoskeletal complications. This comprehensive review synthesizes existing knowledge of microscopic and molecular alterations in CP skeletal muscle. Considerable methodological variability, heterogeneous patient cohorts, and inconsistent control groups significantly complicate comparative interpretations across studies. Nonetheless, some structural abnormalities consistently emerge, including increased variability in muscle fibre size, altered fibre type distribution, long sarcomeres at standardized joint positions, increased collagen content, disrupted neuromuscular junction integrity, reduced capillary density, and mitochondrial and satellite cell impairments. Investigations of satellite cell function in vitro further underscore potential mechanistic alterations, although findings remain inconsistent. Remarkably, few studies have systematically explored the cellular and molecular consequences of standard clinical interventions, revealing a notable research gap. In conclusion, the overall literature reveals considerable divergence in reported outcomes, reflecting the profound complexity of CP muscle biology. We believe that resolving this complexity will require more coordinated and collaborative research approaches.

## Graphical abstract

Cerebral palsy, caused by non‐progressive brain injury, is frequently accompanied by skeletal muscle alterations. This review synthesizes current evidence from muscle biopsy studies, revealing cellular and molecular adaptations in muscle tissue.

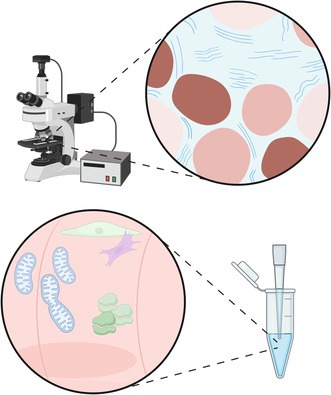

**Abbreviations**
ACL: anterior cruciate ligament
BoNT‐A: botulinum neurotoxin A
CSA: cross‐sectional area
ECM: extracellular matrix
MyHC: myosin heavy chain
NMJ: neuromuscular junction

**What this paper adds**

- Heterogeneous muscle dysfunction in cer‐ ebral palsy is driven by multiple underlying mechanisms.
- Consistent findings include fibre size variability, longer sarcomeres, collagen accumulation, reduced capillary density, and satellite cell and mitochondrial impairments.

Cerebral palsy (CP) is the most common early‐onset neurodevelopmental condition, affecting approximately 1.6 to 3.4 per 1000 live births.^1^, ^2^ It is caused by non‐progressive lesions or malformations in the developing brain, but it is often associated with progressive musculoskeletal complications,^3^ including spasticity,^4^ muscle contractures,^5^ atrophy,^6^ joint deformities,^7^ and reduced mobility.^8^ Children and adults with CP frequently experience muscle weakness,^6^, ^9^ impaired coordination,^10^ increased muscle tone,^11^ and excessive energy expenditure during movement,^12^ all of which contribute to significant limitations in function and quality of life.

Many of these impairments are associated with abnormalities within the skeletal muscle,^13^, ^14^ yet the precise mechanisms remain incompletely understood. While imaging and applied clinical studies have provided important insights, for instance, on muscle volume^15^ or stiffness,^6^, ^16^ microscopic and molecular analysis of human muscle, through biopsies or intraoperative measurements, offers a unique opportunity to investigate the cellular and molecular basis of muscle dysfunction in CP.

This comprehensive review aims to compile and examine all published data derived from biopsies and intraoperative assessments of skeletal muscle in individuals with CP, including, but not limited to, histological, biochemical, biomechanical, molecular, and genetic analyses. By integrating all findings, ranging from muscle fibre microstructure and stiffness, enzymatic functions and gene regulation, to effects of muscle‐ and nerve‐targeted pharmacological interventions, such as botulinum neurotoxin A (BoNT‐A) injections, we aim to clarify current knowledge and identify key gaps in the muscle literature with regard to CP. We have divided the findings from all published papers into seven sections primarily based on physiological domains. The seven sections are skeletal muscle fibres: microstructure and types; intracellular and extracellular muscle stiffness; neuromuscular junction (NMJ); energy metabolism; satellite cells and in vitro experiments; skeletal muscle gene expression profiles; and pharmacological interventions. To support accessibility across disciplines, we present complex findings with conceptual figures and additional explanatory sections throughout. This effort is intended to inform and hopefully facilitate the generation of new ideas in future research on skeletal muscle pathology in CP.

## SKELETAL MUSCLE FIBRES: MICROSTRUCTURE AND TYPES

**Figure 1:**
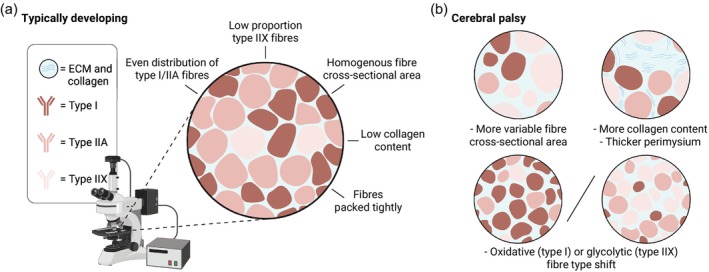
Histological muscle microstructure and fibre types in cerebral palsy. (a) An average histological muscle cross‐section from a typically developing individual. (b) Some of the common histological findings in muscle from individuals with cerebral palsy, including more variable muscle fibre cross‐sectional area, more collagen content, thicker perimysium, and fibre‐type shifts towards either more oxidative type I, or more glycolytic type IIX fibres. Abbreviation: ECM, extracellular matrix.

Skeletal muscle fibre size is commonly assessed using histological examination of muscle biopsies, specifically by measuring the cross‐sectional area (CSA) of fibres. Many studies have examined either total muscle fibre CSA or fibre type‐specific CSA in individuals with CP. A consistent finding across all included studies is the significantly greater variability in muscle fibre CSA observed in individuals with CP compared to typically developing controls (Figure 1).^17^, ^18^, ^19^, ^20^, ^21^, ^22^, ^23^, ^24^, ^25^, ^26^, ^27^, ^28^, ^29^ Two reports further indicated that there may be a positive correlation between the degree of variability in fibre size and functional impairment in CP.^18^, ^21^

Identifying consistent differences in average muscle fibre CSA between the population with CP and typically developing peers has proved challenging. While several studies reported reduced CSA in CP muscles, these results often come with methodological limitations. For instance, some studies have used older control groups,^24^, ^26^, ^30^, ^31^, ^32^ which is known to significantly affect fibre size.^33^ Others measured the CSA of manually dissected muscle fibres,^30^ a technique susceptible to selection bias because thicker fibres are easier to dissect. Also, comparisons across different muscle groups,^28^ each with inherently variable CSA,^34^, ^35^ further complicate the interpretation of these results. On the other hand, two reports that used histochemistry on age‐matched individuals found no difference in muscle fibre CSA between individuals with CP and typically developing peers,^22^, ^29^ while a third observed a larger muscle fibre CSA in the biceps brachii of patients with CP compared to postmortem samples.^27^ Although the notion that decreased muscle fibre CSA in CP aligns well with a decreased muscle mass quantified using ultrasonography,^36^ dual‐energy X‐ray absorptiometry,^37^ and magnetic resonance imaging,^15^, ^38^ so far the results in the literature highlight that established methodological variations in histochemistry and immunohistochemistry techniques,^39^ together with the high variability of fibre sizes in CP, complicate microscopic confirmation of potential fibre size reductions. However, muscle size measured at a macroscopic level may also depend on the number of muscle fibres. Thus, it is possible to have reduced muscle volume without a corresponding reduction in fibre CSA. Nevertheless, as muscle biopsies are predominantly collected from children, careful age matching is crucial to avoid skewing the results or increasing variability. To address the large variability in fibre CSA while simultaneously increasing transparency and resolution of reporting, we suggest using fibre CSA frequency distributions and fibre size cluster analysis in addition to traditional reporting of average fibre CSA.^40^, ^41^ We also suggest that related microstructural outcomes, such as muscle fibre grouping^41^ and fibre‐type shape^42^ be explored further in CP muscle biopsies because this can generate additional insights without further experimental costs.

Reports regarding muscle fibre‐type composition in CP have also produced varied results (Figure 1). For a brief overview of skeletal muscle fibre types, see the section ‘More on skeletal muscle fibre types’ below. Some studies present an increased proportion of fast type IIX fibres,^21^, ^22^, ^23^, ^25^, ^29^, ^31^ whereas others report a larger proportion of slow type I fibres.^19^, ^20^, ^24^, ^28^, ^43^ Adding to this, additional studies have observed either muscle‐dependent differences^17^, ^18^ or no significant changes at all.^26^ Variations in reported fibre‐type data may reflect differences in methodological approaches for fibre typing, the muscles sampled, the level of impairment, the treatment history of the individual, and which muscle was used as control. We hypothesize that changes in fibre‐type distribution in CP may be secondary to alterations in cumulative muscle activation patterns. For instance, decreased usage in a severely contractured forearm muscle may promote a shift towards fast, glycolytic, type IIX fibres,^23^ while increased activation because of persistent muscle tone in ambulatory leg muscles—superimposed on regular activity—might drive a shift towards oxidative type I fibres. Another possibility is that reduced activity pushes muscles towards more myosin type IIX expression, while denervation and atrophy may affect fast‐twitch fibres to a higher degree,^44^ potentially creating a tug‐of‐war situation towards either type I or type IIX predominance depending on the dominant stimulus. Interestingly, similar mechanisms have been proposed to explain the fibre‐type shifts observed in ageing muscle, which is characterized by an overall increase in the proportion of type I fibres, while simultaneously also expressing more type IIX fibres than a younger control group.^41^ Additionally, like ageing muscle, CP muscle displays increased neural cell adhesion molecule‐positive fibres,^20^ which is indicative of denervation.^45^ Supporting this, developmental myosins, that is, embryonic and neonatal myosin heavy chain (MyHC) isoforms, have also been detected at higher concentrations in muscle from children with CP compared to levels commonly observed in typically developing muscle.^21^, ^22^, ^23^ These isoforms are typically expressed only during early development and are gradually replaced by mature MyHC isoforms.^46^ However, their expression can be reactivated in response to cellular insults, such as injury or denervation.

**MORE ON SKELETAL MUSCLE FIBRE TYPES**

Human skeletal muscle fibres are classified into three main types, today most often based on their MyHC isoform content: type I, type IIA, and type IIX.^47^ Historically, fibre types were also distinguished according to their ATPase activity, influencing nomenclature slightly.^48^ Regardless of the identification method, these fibre types exist along a continuum regarding their contractile and metabolic properties: type I fibres exhibit slow‐twitch speed and high oxidative (aerobic) capacity; type IIA fibres display intermediate properties; and type IIX fibres demonstrate a fast‐twitch speed with predominantly glycolytic metabolism (anaerobic).^49^ Fibre‐type distribution varies greatly between individuals. Genetic predisposition explains most of this variation;^50^ however, physiological interventions, such as bed rest or inactivity, promote shifts towards type IIX fibres,^51^ whereas physical exercise training in general drives fibres towards a more oxidative, type I phenotype, with endurance training demonstrating the most pronounced effects.^52^, ^53^, ^54^, ^55^ The latter is especially evident in young, active, typically developing individuals, as type IIX fibres are relatively rare and often undetectable.^39^, ^56^

Fibre‐type distribution also varies substantially between muscles in the same individual. For example, lower body and trunk muscles like the soleus (~90%–70% type I fibres), biceps femoris (~80%–70% type I fibres), and erector spinae (~60% type I fibres) predominantly contain slow, oxidative fibres. Conversely, non‐weight‐bearing muscles with more intermittent activation patterns, such as the triceps brachii (~45%–30% type I fibres), rectus abdominis (~50% type I fibres), and pectoralis major (~50%–40% type I fibres), typically contain a higher proportion of fast‐twitch fibres.^57^, ^58^

**Figure 2:**
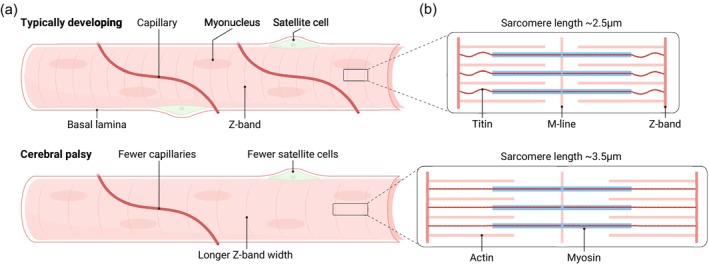
Schematic figure of longitudinal skeletal muscle fibres in individuals with cerebral palsy. (a) Average single muscle fibres from a typically developing individual (top) and an individual with cerebral palsy (bottom). Fewer capillaries and satellite cells, and longer sarcomeres, are often reported. (b) A single sarcomere in typically developing individuals (top) and individuals with cerebral palsy (bottom). Sarcomere lengths are reported to be approximately 40% longer in muscle from individuals with cerebral palsy.

Sarcomeres, the contractile units of skeletal muscle, are aligned longitudinally to form myofibrils within muscle fibres. During maturation and growth, muscles expand longitudinally by the addition of sarcomeres in series, and cross‐sectionally by the addition of new myofibrils.^59^ A consistent microstructural feature of CP muscle with implications for function is increased sarcomere length when contractured muscle is measured at standardized joint angles (Figure 2), documented across both upper‐limb^60^, ^61^ and lower‐limb muscles.^24^, ^31^, ^62^, ^63^, ^64^, ^65^, ^66^ As longer sarcomeres are not accompanied by correspondingly longer actin filaments,^62^ this stretch suggests fewer actin–myosin interactions, adversely affecting muscle force production. Sarcomeres in CP muscles are approximately 40% longer, generating the hypothesis that CP muscles contain fewer sarcomeres in series.^62^ Although challenging to confirm in a definitive manner because entire fibres would need to be extracted, the hypothesis logically aligns with the observation of longer sarcomeres. Notably, sarcomere length correlates positively with both the severity of muscle contractures,^24^, ^61^ Gross Motor Function Classification System levels,^24^ and the degree of hip displacement,^63^ highlighting the potential clinical relevance of serial sarcomere numbers in contracture formation. Despite this evidence, it is currently not known if increased sarcomere length is the cause of contracture formation. For instance, shortening of sarcomeres has been observed in mice subjected to joint fixation using casting,^67^ suggesting that sarcomere length might be a physiological adaptation to contractures.

The underlying pathophysiological mechanisms causing reduced serial sarcomere numbers in CP remain under investigation. Current research has explored possibilities such as reduced numbers, and impaired function of muscle stem cells (satellite cells) and altered muscle gene regulation, both discussed in the coming sections. There are also other molecular regulators of muscle protein turnover that may have a role, some of which have briefly been explored in CP muscle previously, such as increased myostatin (*MSTN*) gene expression and reduced expression of transcription factors regulating ribosome biogenesis.^27^ To date, the number of myonuclei, and several myonuclei‐related mechanisms for growth remain uncharacterized in the context of CP, such as mechanistic target of rapamycin complex 1 (mTORC1), cellular myelocytomatosis (i.e. c‐Myc), androgens, and growth hormones. Moreover, mechanosensing, that is, the ability of the muscle fibre to respond to mechanical stretch, possibly induced by increased muscle tone or extracellular matrix (ECM) stiffness, represents another intriguing avenue for further study.

## INTRACELLULAR AND EXTRACELLULAR MUSCLE STIFFNESS

Muscle stiffness reflects the resistance of muscle tissue to passive lengthening and arises from both intracellular structures, such as cytoskeletal proteins, and extracellular components, primarily the ECM. Muscle stiffness is essential for propulsion, joint stability, and resistance to stretch.^68^ However, in CP, excess muscle stiffness is a well‐recognized clinical feature^6^, ^16^, ^69^ that has been extensively investigated at the cellular and subcellular levels. By comparing the mechanical properties of isolated single muscle fibres and muscle fibre bundles (which include the native ECM), several studies aimed to determine the relative contributions of intracellular versus extracellular components to this increased stiffness; however, the findings remain inconsistent.

Some studies reported that muscle fibres from individuals with CP exhibit increased stiffness at matched sarcomere lengths compared to typically developing controls, implicating intrinsic (intracellular) alterations in passive tension.^30^, ^31^ On the other hand, other investigations found either no difference in fibre stiffness,^24^, ^26^ or the opposite, that is, greater stiffness in typically developing fibres^70^ and myofibrils^64^ at equivalent sarcomere lengths.

Despite these discrepancies, there is general agreement in the literature that muscle fibres from individuals with CP display increased stiffness during ex vivo experiments when assessed at their predicted in vivo sarcomere lengths (~3.5 μm for CP, ~2.5 μm for typically developing individuals).^24^, ^26^, ^30^, ^31^, ^64^ At the level of muscle fibre bundles, which incorporate both muscle cells and the ECM, results are also variable. Some studies demonstrated elevated bundle stiffness in CP muscle,^24^, ^71^ while others found similar^26^, ^31^ or reduced stiffness compared to typically developing bundles,^72^ which makes it difficult to draw any definitive conclusions. These opposing findings have been attributed to potential methodological differences or to the heterogeneity of different muscles from which the bundles were sampled. Regardless, the scattered collective evidence suggests that increased stiffness in CP muscle probably arises from multiple sources rather than a singular pathological mechanism.

Histological observations of increased ECM content in CP muscle date back to Castle et al.^17^ in 1979, with many subsequent studies confirming a marked expansion of the ECM in CP,^24^, ^26^, ^27^, ^72^ particularly within the perimysium (Figure 1).^26^, ^27^ In addition, collagen, the major structural component of the ECM, has been consistently reported to be expressed at a higher degree in CP muscle.^24^, ^27^, ^31^, ^71^, ^73^ Likewise, studies investigating the expression of specific collagen isoforms, as opposed to total collagen content, also reported elevated levels in CP muscle.^71^ The implication of the specific isoforms for clinical function within CP is yet to be determined.^71^ More broadly, the ECM or collagen‐related parameters (such as content or alignment) have been suggested to be of relevance for clinical characteristics such as joint range of motion and hyper‐resistance (e.g. Modified Ashworth Scale), underscoring the potential relevance of ECM remodelling in the functional deficits observed in CP.^66^, ^73^

Within the muscle fibre, titin is the largest known protein (~3800 kDa). It spans from the Z‐disc to the M‐line (Figure 2) and acts like a spring to have a key role in passive elasticity.^74^ A long‐standing hypothesis proposed that a change in titin structure could contribute to increased fibre stiffness in CP.^30^ However, this notion has not been consistently supported because of technical challenges in measuring the molecular weight of titin. One study reported that titin isoforms are heavier in individuals with CP compared to typically developing individuals,^31^ while two other studies found no significant differences.^24^, ^64^ Furthermore, the molecular weight of titin was not associated with passive fibre stiffness.^24^, ^31^ Instead, the titin isoform weight in the gracilis muscle has been positively correlated with hip displacement severity and negatively correlated with sarcomere length,^63^ suggesting that titin may respond adaptively to chronic mechanical changes. While far from conclusive, the findings collectively imply that titin alterations may not be a primary driver of increased muscle stiffness in CP but could represent a compensatory adaptation in response to the muscle shortening associated with contracture formation.

## NEUROMUSCULAR JUNCTION

The NMJ is the specialized synapse at which α‐motor neurons of the peripheral nervous system communicate with skeletal muscle fibres. At the NMJ, acetylcholine is released from the presynaptic motor nerve terminal and binds to nicotinic acetylcholine receptors on the postsynaptic muscle membrane, thereby initiating muscle contraction. To terminate the signal, acetylcholinesterase rapidly hydrolyses acetylcholine within the synaptic cleft, enabling the junction to reset for subsequent activations.^75^

A few studies have examined NMJ structure and molecular composition in the muscles of individuals with CP, comparing the observations to the spinalis muscle of individuals without CP with idiopathic scoliosis.^43^, ^76^, ^77^ Immunofluorescence staining revealed an atypical distribution of nicotinic acetylcholine receptors in CP muscle, with receptors occasionally observed outside the NMJ region, an observation not found in controls.^76^ Quantitative analysis further demonstrated a broader spatial distribution of nicotinic acetylcholine receptors in CP muscle, with greater receptor spread observed in non‐ambulatory compared to ambulatory individuals.^77^

Subsequent work identified the presence of acetylcholinesterase outside the synaptic cleft, signified by acetylcholinesterase staining being scattered beyond the NMJ‐specific protein laminin subunit beta‐2, further indicating synaptic disorganization in CP muscle.^43^ In addition, electron microscopy demonstrated altered structure of the postsynaptic membrane, which is characterized by fewer but deeper junctional folds.^43^ Reduced mitochondrial density within presynaptic terminals was also observed,^43^ which may suggest diminished local ATP production capacity, with potential consequences for neurotransmitter synthesis, vesicle loading, and sustained synaptic transmission.^78^

While the precise functional implications of these NMJ abnormalities remain to be fully elucidated, their presence may reflect a primary consequence of the neurological injury underlying CP, a secondary adaptation to altered neuromuscular activity, or a combination of both.

## ENERGY METABOLISM

In skeletal muscle, the flux of the cellular energy currency ATP can increase 100‐fold from a resting state to maximal exertion.^79^ Because of intermittent and variable energy requirements, skeletal muscles primarily use slow, energetically efficient, aerobic respiration, with faster but less energetically favourable anaerobic glycolysis and creatine kinase breakdown being available during increased ATP demand.^80^ However, to date, skeletal muscle metabolic research in individuals with CP is fairly sparse and has thus primarily investigated aerobic energy pathways.

In an effort to characterize the aerobic energy metabolism in muscles from individuals with CP, reduced capillary density has been highlighted using immunohistochemistry on muscle cross‐sections in two publications.^22^, ^29^ Both upper‐limb^22^ and lower‐limb^29^ muscle biopsies revealed significantly fewer capillaries per muscle fibre, and decreased overall capillary density, suggesting less perfusion both per unit of muscle and per muscle fibre (Figure 2). Of note, reduced capillary density in CP muscle has been observed in diverse cohorts including young adults^22^ and preschool children.^29^ As capillary density has been suggested to be a key factor for muscle fibre growth in an ageing population,^81^ seeing this at such an early age hints at capillaries being a potential overlooked factor in the development of the muscle pathology in CP. However, some contrasting evidence for a reduced capillary density in CP exists. Using fluorescence‐activated cell sorting, which sorts all mononuclear cells, to label and quantify the number of endothelial cells present in the semitendinosus muscle of children with CP, Smith et al.^82^ did not detect a difference between the muscle of individuals with CP and the muscle of typically developing peers. The differences in the molecular identification markers for capillaries used in these studies might account for the differences.

Data on mitochondrial content and function in skeletal muscle of individuals with CP are in line with the findings on capillary density (see the section ‘More on mitochondria’ for a brief introduction to mitochondrial function). Pontén and Stål^22^ reported decreased activity of nicotinamide adenine dinucleotide tetrazolium reductase (complex I) in muscle from individuals with CP, whereas Zogby et al.^32^ found no change in succinate dehydrogenase activity (complex II). Two independent laboratories later published papers indicating mitochondrial impairment in CP due to different and potentially additive underlying mechanisms.^83^, ^84^ Dayanidhi et al.^83^ observed a reduction in enzymatic activity across complexes I, II, and III, but reported no difference in mitochondrial content measures, suggesting impaired mitochondrial function rather than reduced volume. On the other hand, using similar methods (immunoblotting of proteins in complexes I–V), von Walden et al.^84^ observed significant reductions across multiple mitochondrial complexes, suggesting a primary reduction in mitochondrial content. Importantly, some discrepancies may stem from the different control groups used in these papers. Zogby et al.,^32^ Dayanidhi et al.,^83^ and Smith et al.,^82^ focused on endothelial cell fluorescence‐activated cell sorting, used control samples from children undergoing anterior cruciate ligament (ACL) reconstructive surgery, a condition that has since been shown to independently reduce muscle gene and protein expression of mitochondrial enzymes^85^, ^86^ and cause capillary rarefaction^86^ by the time ACL reconstruction is usually scheduled. By contrast, Pontén and Stål^22^ and von Walden et al.^84^ relied on biopsies after accidental death as typically developing controls could potentially affect the comparison to CP differently. Moreover, the former set of studies^32^, ^82^, ^83^ were all conducted in the lower‐limb muscle, whereas the latter set of studies^22^, ^84^ used upper‐limb muscles, which may also partly explain the different results. Notably, electron microscopy‐based assessment of mitochondria in CP muscle has described signs of enlarged, elongated mitochondria with matrix oedema in CP muscles,^20^ but no reduced mitochondrial volume,^87^ which is consistent with an impaired mitochondrial function as speculated by Dayanidhi et al.^83^ Future investigations may consider assessing mitophagy dysfunction, that is, impaired clearance of damaged mitochondria,^88^ because this could reconcile the minor discrepancies between mitochondrial protein levels and functional capacities observed in CP.

Regardless of the underlying mechanisms—volume or function—mitochondrial capacity is diminished in CP muscle.^22^, ^83^, ^84^ This reduction, when combined with the elevated energy cost of movement,^12^ could result in a significant physiological challenge for individuals with CP. These muscle alterations probably create a compound effect: increased energy expenditure of movement, coupled with decreased energy regeneration capacity because of mitochondrial dysfunction, ultimately exaggerate fatigue and limit functional endurance.

**MORE ON MITOCHONDRIA**

The primary function of skeletal muscle mitochondria is to restore ATP to support muscle contraction by oxidizing the carbon molecules obtained from carbohydrates, fats, and proteins. In a series of enzymatic steps within the mitochondrial matrix (Krebs cycle and Beta‐oxidation), long carbon molecules are broken down to carbon dioxide, which is later exhaled, and electrons for the electron transport chain. When oxygen is present within the electron transport chain, electrons flow through a series of large protein complexes, often referred to as complexes I to IV (the exact combination of protein complexes involved in the electron flow depends on the electron's origin). The electron current through complexes I to IV enables complex V to produce ATP.^89^, ^90^ The importance of complexes I to V (often referred to as the oxidative phosphorylation system) for ATP production within the mitochondria, in combination with their ease of quantification, makes them excellent targets in investigations for mitochondrial function within skeletal muscle.

Mitochondria are currently gaining a lot of attention for their role in a vast number of cellular functions besides ATP regeneration, such as acting like cellular signalling processors,^91^ or taking part in cellular anabolism, producing fats, proteins, and nucleotides.^92^ Thus, while the oxidative phosphorylation of protein content and function are often targeted as a proxy for mitochondrial health, several key mitochondrial functions are often overlooked because of the complexity of assessment and the central dogma of mitochondria as the powerhouse of the cell.^93^ It is probably because of its diverse set of functions that mitochondrial capacity is associated with widely diverse outcomes, such as physical fitness,^94^ metabolic^92^ and mental health,^95^ and growth rate in children.^96^

## SATELLITE CELLS AND IN VITRO EXPERIMENTS

Muscle satellite cells are resident stem cells located between the basal lamina and the sarcolemma of muscle fibres. They remain mostly quiescent in adult muscle but are activated in response to injury or stress, where they contribute to muscle development, growth, and repair by fusing into the muscle to serve as myonuclei.^97^ Several studies have reported markedly reduced satellite cell numbers in individuals with CP (Figure 2), both in upper‐extremity^27^ and lower‐extremity muscles.^28^, ^82^, ^98^ These observations have been made using distinct methodologies for quantification, including both immunohistochemistry^27^, ^28^, ^98^ and fluorescence‐activated cell sorting.^82^ However, not all studies have replicated these findings. Two reports—also using immunohistochemistry^29^ and fluorescence‐activated cell sorting^99^—did not observe significant differences in satellite cell abundance between CP and typically developing gastrocnemius muscle. One possible explanation is the use of microbiopsy samples in these latter studies, which provide smaller tissue volumes and may increase variability, especially when quantifying relatively sparse cell populations such as satellite cells.^100^ Although a definitive consensus has not yet been reached, collectively, the literature still supports the notion of an altered satellite cell population in muscle from individuals with CP (Figure 2).

While satellite cells are predominantly quiescent, differences in the proportion of activated, or proliferating, satellite cells may exist between contractured or non‐contractured muscles in individuals with CP.^28^ A possible explanation for altered satellite cell function in contractured muscle is the increased passive mechanical stretch experienced in these muscles.^101^ Passive stretch could desensitize mechanosensing in the satellite cell niche, making them less able to deform in response to stretch and thus leading to an altered function.^102^, ^103^ However, the increased sarcomere length reported in CP did not directly affect the sensitivity of the satellite cell niches in CP muscle as they show similar mechanical deformation of satellite cells as in typically developing children.^101^ This potentially undermines the notion that altered satellite cell activation is a consequence of the mechanical environment in CP muscle. Despite this, it is unclear whether differences within the satellite cell pool are a cause or a consequence of contracture formation. It is certainly compelling to hypothesize that impaired satellite cell activation in specific muscles could contribute to reduced muscle growth and, ultimately, contracture development. While direct evidence is lacking, this hypothesis is based partly on reports suggesting that transgenic mice with reduced satellite cell numbers have an impaired ability to add sarcomeres in series after immobilization‐induced contractures.^67^, ^104^ However, muscle growth can occur, albeit at a blunted level, with significantly reduced satellite cell numbers,^104^, ^105^, ^106^ suggesting that it is not the only mechanism at play. On the other hand, while not strictly required for sarcomerogenesis, satellite cells have a broader role in shaping the muscle microenvironment. They communicate with both fibrogenic cells^107^ and myofibres^108^ to influence ECM remodelling. Thus, disruption of satellite cells in early development could contribute directly to the observed muscle alterations in CP in more ways than stunted sarcomerogenesis.

**Figure 3:**
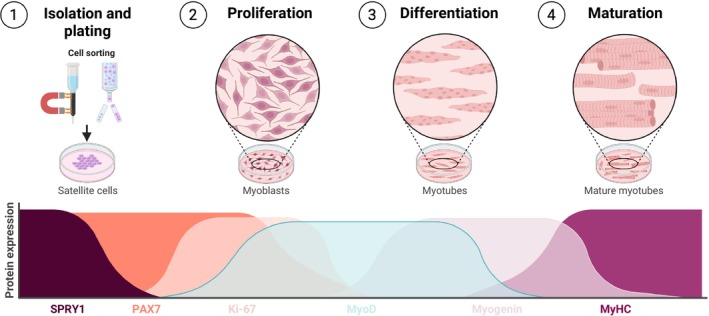
Schematic of different stages of differentiation of myogenic progenitor cells and in vitro experimental workflow. Abbreviations: MyHC: myosin heavy chain; MyoD: myogenic differentiation.

Satellite cells, alongside with other myogenic cells such as fibro‐adipogenic progenitors (interstitial mesenchymal cells that can differentiate into fibroblasts or adipocytes) and mesoangioblasts (vessel‐associated progenitors with myogenic potential) have been isolated from CP muscle and cultured in vitro. For a concise overview of in vitro work using skeletal muscle‐derived progenitor cells, see ‘More on in vitro culture of skeletal muscle‐derived cells’ and Figure 3. Initial investigations reported that satellite cells from CP muscle exhibited increased proliferative activity (hyperproliferation),^109^ while the same cells at later myogenic stages had a reduced differentiating capacity.^109^, ^110^ The hyperproliferative capacity of satellite cells from CP muscle has not received much attention in the literature, with two additional papers reporting proliferating rates to be equal in satellite cells derived from individuals with CP and satellite cells from typically developing individuals.^111^, ^112^ By contrast, the reduced differentiation capacity initially observed^109^, ^110^ has gained further support from two independent research groups.^113^, ^114^ Nevertheless, other reports found contrasting results, either observing no significant differences^115^ or even an increased differentiation capacity in satellite cells from young children with CP compared to typically developing controls.^99^, ^116^ Methodological differences may explain some of these discrepancies. One contributing factor to the opposing results may be that ACL injury, used as a typically developing control model in some studies,^109^, ^110^ may in itself affect satellite cell function and abundance,^117^, ^118^, ^119^ and thus potentially affect comparisons to CP. Another factor may be that studies reporting no difference or increased differentiation potential used microbiopsies for muscle sampling.^99^, ^115^, ^116^ While the smaller biopsy yield obtained from microbiopsies might theoretically increase variability because of lower cell counts, the consistent findings across two reports by Corvelyn et al.^99^, ^116^ suggest that this is unlikely to be a main contributor to the discriminatory results. However, because of the limited amount of tissue, these studies did not perform cell sorting before culture. Consequently, satellite cells were co‐cultured with other myogenic or mesenchymal cell types. While this approach may mimic the physiological environment in which satellite cells reside, it has been highlighted as a potential explanation for the contrasting findings.^120^ Indeed, cross‐talk with fibro‐adipogenic progenitors and immune cells have repeatedly been shown to affect muscle satellite cell function.^121^, ^122^ However, cell–cell interactions have not been extensively studied in muscle from individuals with CP. Thus, rather than representing contradictory findings, we believe these studies instead provide valuable additive insight into the potential effects of co‐culture environments involving satellite cells and other muscle‐derived cells, such as fibro‐adipogenic progenitors and mesoangioblasts.^99^, ^115^, ^116^

Besides characterizing the intrinsic properties of satellite cells, in vitro experiments have also provided insights into the molecular underpinnings of muscle dysfunction in CP. For instance, one study reported that the stiffness of the plating milieu had minimal effect on the capability of satellite cells from either CP or typically developing skeletal muscles to differentiate into myotubes.^114^ On the other hand, fibro‐adipogenic progenitors isolated from CP muscle exhibited an increased propensity for fibrotic activation and reduced sensitivity to the surrounding stiffness relative to fibro‐adipogenic progenitors from typically developing individuals.^114^ This may suggest a compromised negative feedback loop for collagen formation in the ECM in CP muscle. In another study, the lower differentiation capacity of myoblasts derived from CP muscle was mechanistically linked to lower levels of circular RNA derived from the nuclear factor IX.^113^ Lower circular RNA nuclear factor IX levels were associated with elevated microRNA‐373‐3p, which in turn reduced the expression of the transcription factor MEF2C,^113^ an important regulator of key myogenic genes.^113^, ^123^, ^124^ Other reported findings from cell culture experiments include a relatively small difference in the transcriptional landscape when comparing myotubes derived from CP muscle and from the muscle of typically developing individuals, mainly patients with scoliosis, with significant differences in only 90 of approximately 7500 identified genes.^111^

Epigenetic mechanisms, including histone modifications and DNA methylation, distinctly regulate quiescence, proliferation, and differentiation of myogenic cells.^125^ The latter, DNA methylation, has been implicated in contributing to the impaired capacity of myogenic cells isolated from CP muscle to differentiate. Specifically, Domenighetti et al.^110^ and Sibley et al.^109^ linked the reduced differentiation capacity of CP‐derived myocytes to the increased methylation levels of myogenesis‐related genes. Contrasting these data, Robinson et al.^112^ described a general reduction in the degree of DNA methylation in proliferating myoblasts from children with CP and cells from aged‐matched patients with scoliosis and ACL undergoing surgery,^112^ suggesting context‐dependent methylation dynamics.

**MORE ON IN VITRO CULTURE OF SKELETAL MUSCLE‐DERIVED CELLS**

Skeletal muscle tissue contains multiple cell populations with regenerative or regulatory capacity. In vitro studies often isolate and expand these cells to investigate their molecular characteristics and functional potential under controlled conditions. Among these, satellite cells are of particular interest because they represent the muscle's resident stem cells.^97^ Satellite cell culture enables researchers to model key stages of the muscle‐forming (myogenic) process (Figure 3). The major phases typically observed are: (1) isolation and plating: satellite cells are isolated from muscle biopsies, often through enzymatic digestion and mechanical dissociation. They are then plated on coated surfaces (e.g. collagen or Matrigel) to support attachment and growth. At this stage, cells typically express the stem cell marker PAX7, a hallmark of quiescent and early activated satellite cells; (2) activation and proliferation (myoblast stage): once in culture, satellite cells become activated and transition into proliferating myoblasts, that is, committed muscle precursors. When cultured in growth medium rich in serum and nutrients, myoblasts divide rapidly to expand the population. This phase is marked by the expression of MyoD, alongside PAX7, and proliferation is commonly measured according to doubling time, the period required for the cell number to double; (3) differentiation and fusion (myotube stage): switching to a serum‐poor differentiation medium triggers myoblasts to exit the cell cycle and start differentiating. Cells elongate and fuse into multinucleated myotubes, that is, early muscle fibres. Key markers include myogenin and embryonic MyHC. The fusion index is used to quantify the extent of cell fusion by calculating the percentage of nuclei contained within myotubes; and (4) maturation: myotubes eventually mature into structures that resemble functional muscle fibres; This stage is characterized by the expression of mature MyHC isoforms and the assembly of sarcomere structures. Typical assessments at this stage include quantifying mature myosin expression, sarcomere organization, metabolic markers, and contractile functionality, depending on the aim of the study.^126^

## SKELETAL MUSCLE GENE EXPRESSION PROFILES

In addition to gene regulation in satellite cells, several gene expression analyses and investigations into the translational and gene‐regulating machinery have been conducted in bulk skeletal muscle tissue. Diverse methods have been used, including comprehensive microarrays,^127^, ^128^ bulk RNA sequencing,^111^ smaller custom gene panels,^65^, ^87^ and targeted quantitative polymerase chain reaction.^27^, ^43^, ^76^, ^84^ The breadth of these data sets varies significantly in terms of the magnitude of differences between CP and typically developing muscle. For example, an initial microarray analysis identified approximately 200 differentially expressed genes in the forearm muscles of six children with CP,^127^ while a subsequent larger study identified around 1400 differentially expressed genes in a knee flexor muscle of children with CP versus patients undergoing ACL surgery.^128^ In contrast, RNA sequencing comparisons of erector spinae muscles from patients with CP and patients with idiopathic scoliosis, reported only 87 differentially expressed genes, representing just 1.4% of detected genes.^111^ Smaller targeted panels reported divergent results: Pingel et al.^65^ identified differential expression in approximately half of the 92 genes studied between children with CP and typically developing controls, while the same panel detected only 14 differentially expressed genes when comparing adults with CP with typically developing adults.^87^ The reasons for these discrepancies are not clear but may be attributed to age or other underlying patient‐specific factors.

Several gene expression signatures across these data sets align with previously reported findings in CP muscle. For instance, increased expression of collagen and ECM‐related genes has been frequently noted,^27^, ^127^, ^128^ sometimes even correlating with altered mechanical muscle properties such as increased stiffness.^128^ However, results are not universally consistent, with some studies observing no significant differential regulation of ECM‐associated genes or mixed patterns of upregulation and downregulation.^65^, ^111^ Similarly, multiple reports consistently indicate a downregulation of genes related to mitochondrial function and energy metabolism across various methodologies.^65^, ^84^, ^87^, ^127^, ^128^ In contrast, gene expression changes related to the muscle contraction machinery and cytoskeletal elements do not exhibit a clear directional trend, with both overexpression and underexpression of certain genes reported. Likewise, inflammatory markers show variable expression within and between studies, although targeted analyses strengthen the evidence for elevated pro‐inflammatory cytokines in CP muscle.^27^

Interestingly, increased expression of the transcription factor Myc, implicated in ribosomal biogenesis and fibre‐type specification,^129^ was reported in two data sets examining lower‐limb muscle from individuals with CP.^65^, ^87^ Myc elevation, known to promote a slow‐twitch fibre phenotype in mice, may explain part of this shift in specific muscles. However, an increased Myc expression across all muscles seems paradoxical given the reported reductions in ribosomal RNA in CP muscles.^27^ These discrepancies may reflect muscle‐specific differences, given that upper‐limb muscles, which were assessed for ribosomal RNA content, frequently exhibit shifts towards faster type IIX fibres.^27^

Another notable observation concerns MEF2C, discussed in an earlier section. Unlike findings in satellite cell‐derived myoblasts indicating reduced expression,^113^ mature muscle from patients with CP exhibited increased MEF2C expression.^111^ This may indicate an active regeneration process or heightened proliferative activity, which is consistent with elevated expression of markers like neural cell adhesion molecule and embryonic myosin observed in CP muscle.

## PHARMACOLOGICAL INTERVENTIONS

Few strategies are available for managing spasticity in individuals with CP, with the most commonly used being local injections of BoNT‐A. Initially introduced in the early 1990s, BoNT‐A has since become a standard intervention for reducing muscle spasticity in children with CP.^130^ The mechanism involves inhibiting acetylcholine release at the NMJ, leading to chemical denervation and temporary muscle relaxation.^131^ While the short‐term benefits of BoNT‐A are documented (months after injection), including reductions in spasticity and improvements in gait and function,^132^ its long‐term impact on skeletal muscle health and integrity remains a matter of investigation.

Preclinical animal studies demonstrated histopathological alterations and muscle atrophy after BoNT‐A exposure;^133^, ^134^ signs of neurogenic atrophy have been observed in adult typically developing muscle up to a year after injection.^135^ Despite widespread clinical use, investigations of the effects of BoNT‐A on the skeletal muscle of children with CP are limited. Moreover, variability in the muscle group examined, dosing regimen, injection frequency, outcome measures, and participant characteristics complicates interpretation and comparisons across studies.

In one study, gastrocnemius muscle samples collected from children with CP (mean age 11 years) who had received BoNT‐A between 3 months and 9 years before biopsy sampling were compared to untreated vastus lateralis muscle from the same individuals.^136^ Findings included a reduced proportion of type I fibres and features of mild neurogenic atrophy.^136^ On the other hand, upper‐limb muscles treated with BoNT‐A in a similarly aged cohort displayed increased muscle fibre CSA and greater capillary density compared to untreated controls. However, elevated neonatal MyHC expression and centrally located nuclei were also present, thus reflecting ongoing cycles of degeneration–regeneration.^137^ As both investigations relied on cross‐sectional comparisons, causal interpretation remains limited.

To address this, a 3‐ to 6‐month follow‐up study in children aged 2 to 8 years was conducted on the medial gastrocnemius. While some signs of fibre atrophy were observed 3 months after injection, followed by larger sizes at 6 months, these effects were not significant. Instead, increased variability in fibre size and a shift towards a more oxidative phenotype persisted, indicating potentially long‐lasting alterations.^138^ Subsequent in vitro work from the same laboratory showed that while direct BoNT‐A exposure did not affect myogenic differentiation, impaired fusion capacity was evident in cells derived from muscle biopsies obtained 6 months after BoNT‐A administration.^115^ These findings suggest a possible long‐term imprint also on satellite cells that could compromise future regenerative responses.

Beyond BoNT‐A, other pharmacological strategies are being explored. A recent proof‐of‐concept study demonstrated that ex vivo collagenase treatment reduced collagen content and decreased passive stiffness in muscle fibre bundles from the adductor longus of children with CP.^139^ While these findings are promising, the safety and efficacy of such approaches must be determined.

In summary, the literature on the long‐term effects of BoNT‐A in the skeletal muscle of individuals with CP remains inconsistent, probably because of substantial variation in study design, treatment protocols, treated muscle group, and patient characteristics. Despite this, reports raise concerns regarding adverse impacts on muscle microstructural and regenerative potential. Persistent myocellular alterations, including disrupted satellite cell behaviour, may extend beyond the resolution of acute atrophy. These findings underscore the need for cautious clinical application of BoNT‐A and support the development of optimized treatment protocols, exploring variables such as injection intervals, synergetic physiotherapy, and other additive muscle preservation strategies.

## METHODOLOGICAL CONSIDERATIONS AND FUTURE DIRECTIONS

Conducting invasive research using muscle biopsies in paediatric populations, particularly in children with CP, poses several significant challenges. First, CP is inherently heterogeneous, characterized by a wide spectrum of clinical presentations and diverse aetiological insults.^140^ This manifests in a large variability in muscle characteristics within and between individuals with CP. Such variability probably underlies distinct molecular mechanisms contributing to muscle pathology either directly or by affecting confounders such as physical activity levels, thus complicating generalizations across studies. Second, muscle biopsies from children with CP are typically obtained during surgical interventions, such as orthopaedic corrections or tendon lengthening procedures. These muscles are rarely treatment‐naive, often reflecting varied rehabilitation histories and pharmacological interventions, including BoNT‐A injections, serial casting, and intensive physiotherapy. Consequently, biopsies inherently carry heterogeneous treatment backgrounds, affecting their biological characteristics. Third, differences in the sampled muscle groups across studies add another layer of complexity. Skeletal muscle microstructure^57^ and molecular signatures^141^ vary significantly between muscle groups. Thus, comparing molecular and microstructural data derived from different muscles, such as the forearm versus the leg, can be inherently problematic and may lead to confounded conclusions. Additionally, the selection of suitable control samples from typically developing children introduces further confounding factors. As skeletal muscle biopsies from typically developing children are extremely rare, control biopsies are often sampled from children who have sustained a trauma, such as a fracture or an ACL disruption, or have idiopathic scoliosis, where as yet we do not know the cause. While these controls may accurately reflect certain measures, they might inadvertently mask abnormalities in other parameters. Moreover, precise age matching between the group with CP and the typically developing group is critical, particularly for microstructural comparisons. Considering that skeletal muscle mass increases substantially (~500%) from early childhood to adolescence,^38^, ^142^ even slight age discrepancies may significantly confound outcomes. Therefore, careful age matching is essential and unmatched age comparisons should be approached with considerable caution.

To mitigate these challenges, coordinated and methodologically rigorous collaboration is essential. Multicentre partnerships can reduce implicit biases inherent in single‐site workflows, enable standardized inclusion and exclusion criteria, and facilitate exchange of control tissue, ensuring that findings are not dependent on any single typically developing comparison cohort. Similarly, sharing technical expertise across laboratories can expand the scope of questions addressed from each biopsy. An equally important step is routine deposition of raw data alongside publications, which enhances transparency, enables secondary analyses, and reduces the need for repeated tissue sampling to answer adjacent questions. While a few data sets of individuals with CP and typically developing peers have followed these practices to some extent,^25^, ^127^, ^128^, ^143^ broader adoption, either as supplementary files, links to curated repositories, or formal data resources, would accelerate progress and reproducibility in the field.

## CONCLUSION

Although studies using skeletal muscle biopsies in CP have substantially increased in recent years, significant gaps in understanding remain. Merely delineating the skeletal muscle phenotype in CP has proven exceedingly complex, largely because of the inherent aetiological and phenotypic heterogeneity of the disorder. Consequently, reliance on a single publication to substantiate specific phenotypic features in CP muscle is inadvisable. Indeed, current literature demonstrates contradictory findings, variously supporting opposing shifts in fibre‐type composition, disparate ECM stiffness, inconsistent mitochondrial volumes, and variable expression of critical regulatory genes. Moreover, many studies report null findings, further complicating the interpretive landscape. Despite these inconsistencies, certain skeletal muscle characteristics in CP are more reliably documented across multiple studies. Notably, increased variability in muscle fibre CSA, altered fibre‐type distribution, longer sarcomeres, increased collagen content, disrupted NMJ integrity, diminished aerobic capacity and reduced capillarization, and an altered—potentially impaired—satellite cell population emerge as relatively consistent phenotypic hallmarks. At a more fundamental mechanistic level, the precise drivers underpinning the progressive deterioration observed in CP skeletal muscle remain largely unresolved. Preliminary investigations suggested intriguing roles for epigenetic modifications and aberrant back‐splicing within satellite cells, yet these hypotheses necessitate further validation through diverse methodological approaches, involving more muscle groups and additional typically developing control cohorts, before being considered conclusive. Beyond phenotype characterization and initial mechanistic insights, surprisingly few biopsy studies have systematically assessed the molecular and structural impact of therapeutic standard of care such as pharmacological interventions, orthopaedic surgery, or exercise training interventions. Therefore, it is imperative that future research expands to address these therapeutic effects rigorously. Enhanced collaborative efforts across laboratories and institutions, leveraging diverse patient populations and specialized analytical expertise, will be indispensable for resolving the profound heterogeneity inherent in CP muscle research. Finally, promoting open access to raw data repositories will significantly bolster hypothesis generation and methodological transparency, ultimately advancing the field.

## CONFLICT OF INTEREST STATEMENT

The authors have stated that they had no interests which might be perceived as posing a conflict or bias.

## Data Availability

Data sharing not applicable ‐ no new data generated.
